# CSF biomarkers of reactive glial cells are associated with blood–brain barrier leakage and white matter lesions

**DOI:** 10.1186/s40035-024-00422-z

**Published:** 2024-05-23

**Authors:** Linbin Dai, Xinyi Lv, Zhaozhao Cheng, Yan Wu, Xianliang Chai, Jiong Shi, Yong Shen, Qiong Wang, Feng Gao

**Affiliations:** 1 Department of Neurology, The First Affiliated Hospital of USTC, Division of Life Sciences and Medicine, University of Science and Technology of China, Hefei, 230027 China; 2 Institute On Aging and Brain Disorders, The First Affiliated Hospital of USTC, Division of Life Sciences and Medicine, University of Science and Technology of China, Hefei, 230027 China; 3 Center for Excellence in Brain Science and Intelligence Technology, Chinese Academy of Sciences, Shanghai, 230027 China; 4 Huang Shan Road 443, Hefei, 230027 Anhui China

## Main text

Cerebral small vessel disease (CSVD) is a prevalent cerebrovascular disease characterized by chronic vascular dysfunction [1], primarily diagnosed using MRI-based markers such as white matter hyperintensities (WMHs), cerebral microbleeds, perivascular spaces, and lacunes [2]. CSVD also involves blood–brain barrier (BBB) disruption, evident with an elevated cerebrospinal fluid (CSF)/serum albumin ratio (also called Albumin Quotient, QAlb) [3]. Despite these markers reflecting different aspects of cerebrovascular disease, its underlying causes are not fully understood. Interestingly, CSVD often coincides with Alzheimer's disease (AD) pathology [4], and abnormal tau pathology affects brain vessel architecture and worsens white matter neurite density [5]. More importantly, glial cell-mediated neuroinflammation is involved in the onset and progression of both CSVD and AD [6]. However, it is unclear whether the contribution of neuroinflammation to cerebrovascular injury is independent of AD pathology and the association between CSF biomarkers of reactive glial cells and CSVD features remains unknown.

In this study, we included 52 cognitively unimpaired individuals, 42 patients with mild cognitive impairment, 75 patients with AD, and 27 participants with non-AD dementia. There were no significant differences in sex among the clinical groups. The demographic characteristics are summarized in Additional file 1: Table S1. Neuroinflammatory markers in CSF were analyzed, including MIF, CCL-2, CXCL-8, YKL-40, S100B, and LCN2. BBB permeability was assessed using the QAlb method, and the extent of CSVD was measured using MRI (Additional file 2: Supplementary Methods).

**Fig. 1:**
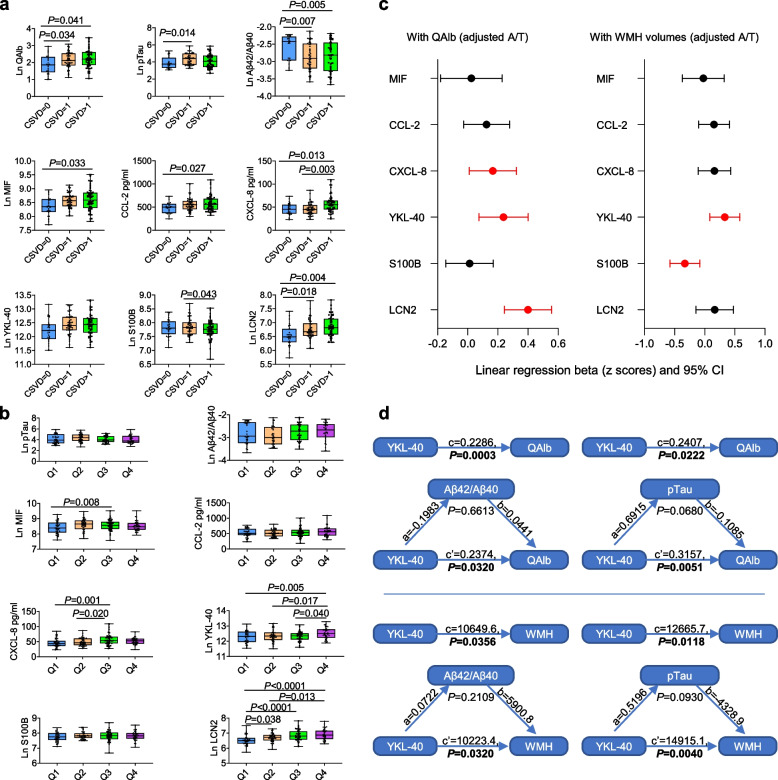
Levels of CSF biomarkers in different groups and their associations with cerebrovascular damage features. **a, b** Levels of CSF biomarkers across CSVD burdens (**a**) and QAlb stage (**b**). The box plots depict the median (horizontal bar), interquartile range (IQR, hinges), and the whiskers indicate the minimum and maximum values. (Quartile of Ln QAlb: Q1: 1.0–1.81; Q2: 1.81–2.13; Q3: 2.13–2.46; Q4: 2.46–3.46). *P* values were assessed by a one-way analysis of covariance (ANCOVA) adjusted by age, sex, *APOE*-ε4*,* Aβ42/Aβ40, and pTau. **c** Associations between different neuroinflammatory markers and cerebrovascular damage summarized in forest plots. Linear regression models were adjusted by age, sex, *APOE*-*ε4*, Aβ42/Aβ40, and pTau. (The red line represents *P* < 0.05, the black line represents *P* > 0.05). **d** Mediation analysis of YKL 40 alteration affects the BBB damage and white matter lesions. Mediation analysis included the following variables: Aβ42/Aβ40 or pTau were treated as a mediator, QAlb and WMH volumes were set as the dependent variable, and YKL-40 was set as the independent variable. Analyses based on multiple linear regression models with sex, age, and *APOE*-ε4 adjusted as covariates. *P* < 0.05 was considered statistically significant

The levels of CSF neuroinflammatory markers were analyzed in individuals with different CSVD burdens (Fig. 1a). Elevated pTau levels were found in mild CSVD (CSVD = 1, *P* = 0.014) cases, while decreased CSF Aβ42/Aβ40 ratios were observed in patients with mild (*P* = 0.007) or severe CSVD (CSVD > 1, *P* = 0.005). To rule out the potential effects of AD pathology, CSF Aβ42/Aβ40 and pTau were adjusted as covariates. Compared to controls, CSVD participants exhibited higher QAlb (CSVD = 1: *P* = 0.034; CSVD > 1: *P* = 0.041) and LCN2 (CSVD = 1: *P* = 0.018; CSVD > 1: *P* = 0.004) levels. MIF (*P* = 0.033) and CCL-2 (*P* = 0.027) levels were elevated only in severe CSVD cases. CXCL-8 levels were higher in severe CSVD compared to controls (*P* = 0.013) and mild CSVD (*P* = 0.003), while severe CSVD patients had lower S100B levels than mild CSVD cases (*P* = 0.043). CSF neuroinflammatory marker levels in different groups defined by other CSVD features were also analyzed (Additional file 1: Figs. S1-S12).

We then evaluated the levels of neuroinflammatory markers at various stages of BBB damage. The QAlb values were divided into quartiles to assess the extent of BBB damage: Q1 (no damage), Q2 (mild damage), Q3 (moderate damage), and Q4 (severe damage) (Fig. 1b). Interestingly, AD core biomarkers (pTau and Aβ42/Aβ40), CCL-2, and S100B showed minimal changes across QAlb quartiles. MIF (*P* = 0.008) and CXCL-8 (*P* = 0.001) increased at moderate BBB damage but tended to decrease at severe BBB dysfunction. Patients with severe BBB dysfunction had the highest YKL-40 levels (Q4 vs. Q1, *P* = 0.005; Q4 vs. Q2, *P* = 0.017; Q3 vs. Q3, *P* = 0.040). LCN2 increased significantly from early BBB damage (Q2 vs. Q1, *P* = 0.038), peaking at the severe stage (Q4 vs. Q1, *P* < 0.0001; Q4 vs. Q2, *P* = 0.013).

Subsequently, we performed univariate linear regression to explore associations of neuroinflammatory markers with QAlb and WMH volumes. Elevated QAlb correlated with increased CXCL-8 (β = 0.166, *P* = 0.038), YKL-40 (β = 0.238, *P* = 0.005), and LCN2 (β = 0.400, *P* < 0.0001). Higher WMH volumes were associated with elevated YKL-40 (β = 0.333, *P* = 0.010) but decreased S100B (β =  − 0.329, *P* = 0.010) (Fig. 1c and Additional file 1: Table S2). These associations remained significant even without adjusting for CSF Aβ42/Aβ40 and pTau (Additional file 1: Fig. S13). AD patients exhibited different association patterns compared to non-AD groups (Additional file 1: Table S3).

To assess the contribution of CSF neuroinflammatory markers to QAlb and WMH volumes, we conducted multivariate analysis, incorporating significant markers identified from univariate analyses. Covariates included age, sex, *APOE-*ε4, Aβ42/Aβ40, and pTau. The *R*^2^ values for the models with markers alone and markers in combination with covariates are summarized in Additional file 1: Table S4. While covariates significantly contributed to higher QAlb (*R*^2^ = 0.080, *P* = 0.003), regression values for QAlb were similar between models with markers alone and with markers + covariates (*R*^2^ = 0.314 versus *R*^2^ = 0.366), suggesting neuroinflammation is a primary mediator of the effect on QAlb. For WMH volumes, models with YKL-40 and S100B remained significant (*R*^2^ = 0.334, *P* = 0.002), whereas covariates did not (*R*^2^ = 0.089, *P* = 0.071). Notably, YKL-40 directly contributed to BBB damage and WMH lesions independent of AD pathologies (Fig. 1d).

Although the involvement of inflammation in CSVD is well recognized [7], the relationship of neuroinflammation, particularly CSF neuroinflammatory markers closely related to glial cells, with cerebrovascular dysfunction, remains largely unexplored. Our study revealed associations between CSF neuroinflammatory markers closely related to glial cells (YKL-40, S100B, and LCN2) and cerebrovascular injury. YKL-40 positively correlated with both QAlb and WMH volumes, implicating its role in BBB permeability and WMH progression. Conversely, elevated LCN2 levels were linked to worsened BBB permeability, while S100B negatively affected WMH volumes.

A large proportion of AD patients exhibit cerebrovascular dysfunction, complicating the understanding of the role played by neuroinflammation in mixed AD and cerebrovascular disease. Recent positron emission tomography studies revealed distinct pathways of neuroinflammation and Aβ deposition, independently contributing to the progression of mixed AD and vascular dementia pathologies [8]. Similarly, our data showed increased glial cell-associated neuroinflammatory markers during early CSVD and BBB damage stages, even after adjustment for AD-related pathologies. This highlights the glial cell-associated neuroinflammation as an early event in cerebrovascular disease.

Our study has several limitations. First, it was cross-sectional and conducted in a single center, limiting the generalizability to other regions, especially in a vast country like China. Second, we did not measure CSF biomarkers of vascular inflammation secreted by activated endothelial cells, which are crucial components of the BBB and vasculature. Future research should explore the causal relationship and sequence of vascular inflammation and pathophysiological events. Furthermore, lipids and metabolites that contribute to vascular brain injury should also be considered in future investigations. Last, we did not include the analysis of soluble PDGFRβ (sPDGFRβ) in this study. PDGFRβ is a type of tyrosine kinase receptor expressed by pericytes, and sPDGFRβ in the CSF has been suggested to be closely associated with pericyte and BBB damage [9]. In particular, a recent study has shown that high baseline levels of CSF sPDGFRβ predict the future cognitive decline in carriers of *APOE4* gene, a major risk factor for AD [10]. Therefore, it would be important to investigate pericyte-related biomarkers to understand the pathophysiology of neurodegenerative diseases, particularly in relation to cerebrovascular dysfunction.

In summary, our findings provide evidence that neuroinflammatory CSF biomarkers tightly related to glial cells play a significant role in distinct pathological processes associated with cerebrovascular damage, which is independent of AD pathologies. Future studies are necessary to fully understand the functional profiles of specific neuroinflammatory markers in cerebrovascular dysfunction. Such investigations would not only aid in the identification of monitoring biomarkers but also facilitate the development of targeted therapeutic strategies.

### Supplementary Information

**Additional file 1:** **Fig. S1** Levels of fluid biomarkers in different groups defined by WMH status. **Fig. S2** Levels of fluid biomarkers in different groups defined by Aβ and WMH status. **Fig. S3** Levels of fluid biomarkers in different groups defined by tau and WMH status. **Fig. S4** Levels of fluid biomarkers in different groups defined by CMB status. **Fig. S5** Levels of fluid biomarkers in different groups defined by Aβ and CMB status. **Fig. S6** Levels of fluid biomarkers in different groups defined by tau and CMB status. **Fig. S7** Levels of fluid biomarkers in different groups defined by PVS status. **Fig. S8** Levels of fluid biomarkers in different groups defined by Aβ and PVS status. **Fig. S9** Levels of fluid biomarkers in different groups defined by tau and PVS status. **Fig. S10** Levels of fluid biomarkers in different groups defined by lacunes status. **Fig. S11** Levels of fluid biomarkers in different groups defined by Aβ and lacunes status. **Fig. S12** Levels of fluid biomarkers in different groups defined by tau and lacunes status. **Fig. S13** Associations between CSF neuroinflammatory markers and cerebrovascular damage summarized in forest plots. **Table S1**. Demographic characteristics of the CANDI cohort. **Table S2**. Associations between features of cerebrovascular damage and neuroinflammation markers. **Table S3**. Associations between features of cerebrovascular damage and neuroinflammation markers in AD and non-AD (including CU, MCI and Non-ADD) groups. **Table S4**. R^2^ in models for separate markers significantly associated with QAlb and WMH volumes.

**Additional file 2.** Supplementary Methods.

## Data Availability

The data that support the findings of this study are available from the corresponding authors upon reasonable request.

## Abbreviations

CSVD: Cerebral small vessel disease
AD: Alzheimer’s disease
CSF: Cerebrospinal fluid
QAlb: CSF/plasma albumin quotient
WMHs: White matter hyperintensities
BBB: Blood–brain barrier
