# Supplementary material for: CSF biomarkers of reactive glial cells are associated with blood–brain barrier leakage and white matter lesions
Source: Transl Neurodegener. 2024 May 23;13:26. doi: 10.1186/s40035-024-00422-z (PMC11112808; doi:10.1186/s40035-024-00422-z)
Supplement: Supplementary file 1 — Additional file 1: Fig. S1 Levels of fluid biomarkers in different groups defined by WMH status. Fig. S2 Levels of fluid biomarkers in different groups defined by Aβ and WMH status. Fig. S3 Levels of fluid biomarkers in different groups defined by tau and WMH status. Fig. S4 Levels of fluid biomarkers in different groups defined by CMB status. Fig. S5 Levels of fluid biomarkers in different groups defined by Aβ and CMB status. Fig. S6 Levels of fluid biomarkers in different groups defined by tau and CMB status. Fig. S7 Levels of fluid biomarkers in different groups defined by PVS status. Fig. S8 Levels of fluid biomarkers in different groups defined by Aβ and PVS status. Fig. S9 Levels of fluid biomarkers in different groups defined by tau and PVS status. Fig. S10 Levels of fluid biomarkers in different groups defined by lacunes status. Fig. S11 Levels of fluid biomarkers in different groups defined by Aβ and lacunes status. Fig. S12 Levels of fluid biomarkers in different groups defined by tau and lacunes status. Fig. S13 Associations between CSF neuroinflammatory markers and cerebrovascular damage summarized in forest plots. Table S1. Demographic characteristics of the CANDI cohort. Table S2. Associations between features of cerebrovascular damage and neuroinflammation markers. Table S3. Associations between features of cerebrovascular damage and neuroinflammation markers in AD and non-AD (including CU, MCI and Non-ADD) groups. Table S4. R2 in models for separate markers significantly associated with QAlb and WMH volumes. [file 40035_2024_422_MOESM1_ESM.pdf]

CSF markers of reactive astrocytes associated with blood–brain barrier leakage and white matter lesions independent of Alzheimer's disease pathologies

Supplementay figure

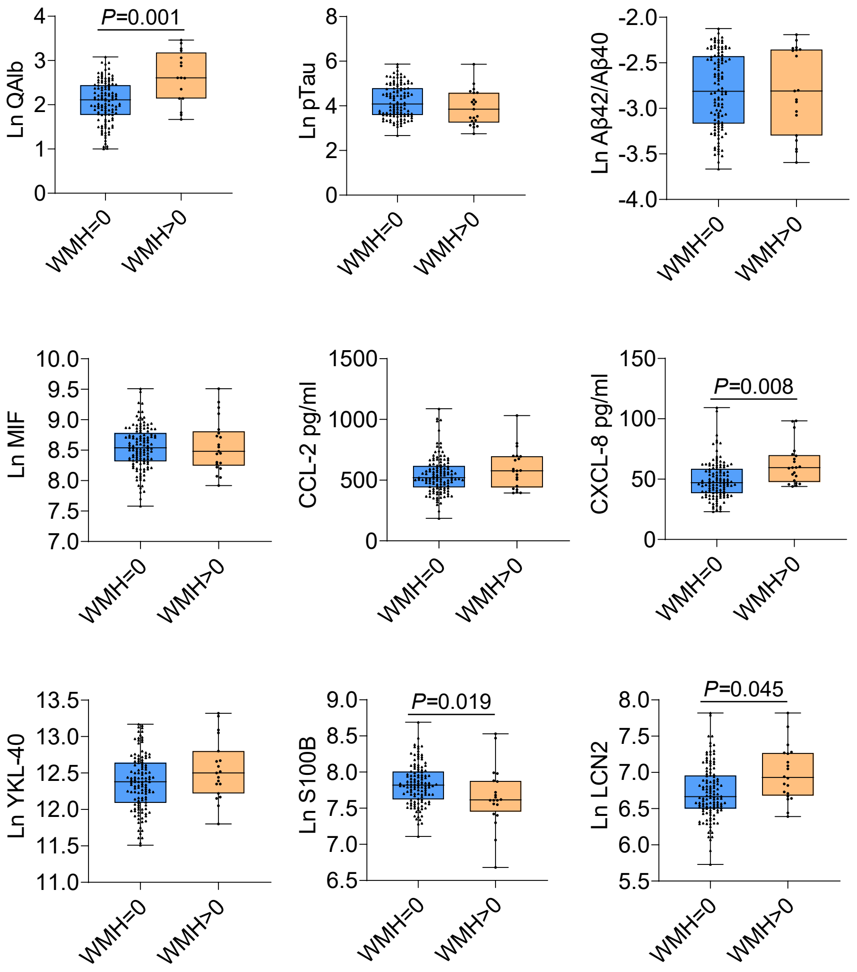

Fig.S1 Levels of fluid biomarkers in different groups defined by WMH (white matter hyperintensities) status. The box plots depict the median (horizontal bar), interquartile range (IQR, hinges), and the whiskers indicate the minimum and maximum values. *P*-values were assessed by a one-way analysis of covariance (ANCOVA) adjusted by age, sex, *APOE-ε4*, Aβ42/Aβ40, and pTau.

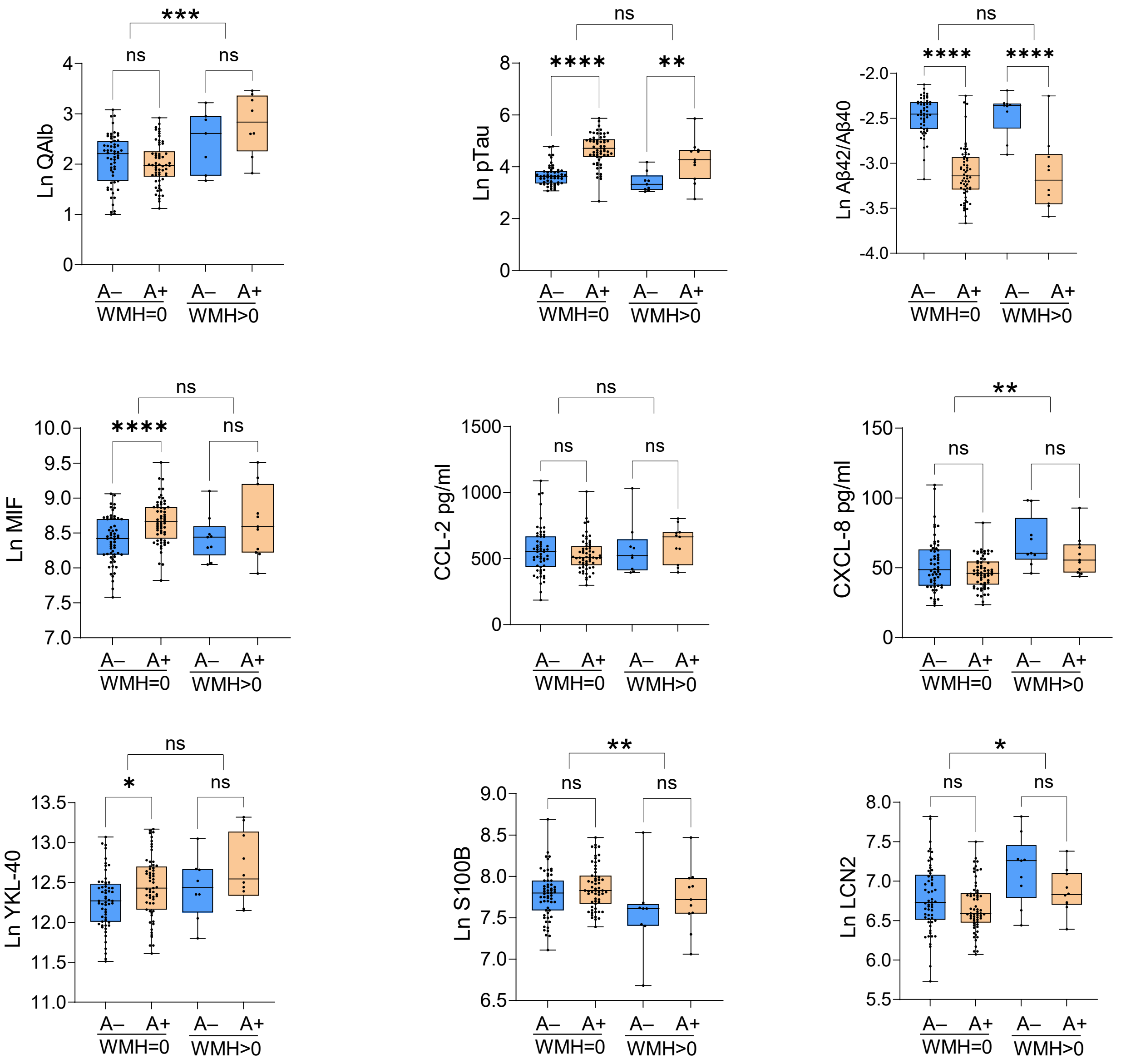

Fig.S2 Levels of fluid biomarkers in different groups defined by Aβ and WMH (white matter hyperintensities) status. The box plots depict the median (horizontal bar), interquartile range (IQR, hinges), and the whiskers indicate the minimum and maximum values. *P*-values were assessed by a one-way analysis of covariance (ANCOVA) adjusted by age, sex and *APOE-ε4.* *P<0.05, **P<0.01, ***P<0.001, ****P<0.0001.

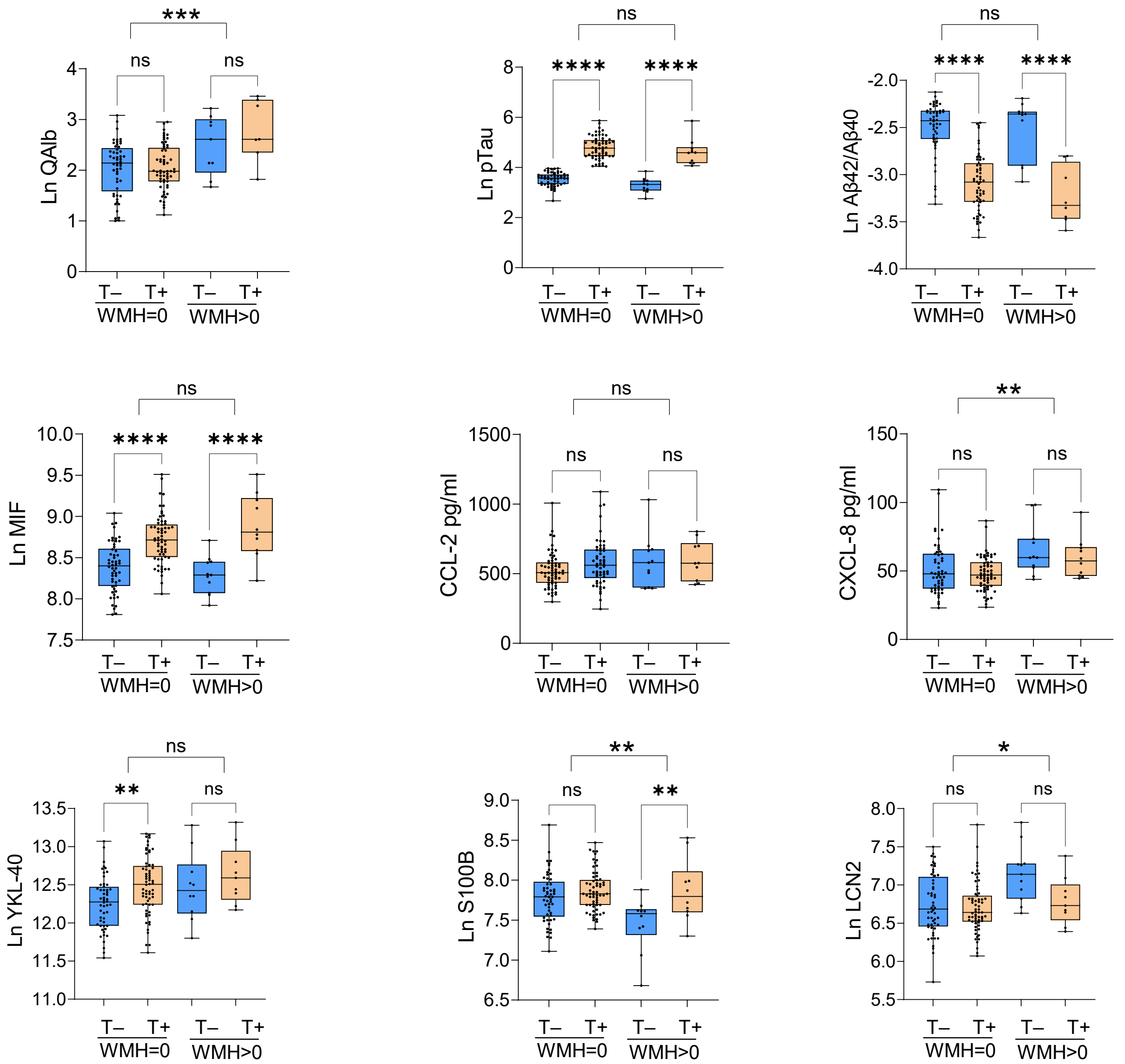

Fig.S3 Levels of fluid biomarkers in different groups defined by tau and WMH (white matter hyperintensities) status. The box plots depict the median (horizontal bar), interquartile range (IQR, hinges), and the whiskers indicate the minimum and maximum values. *P*-values were assessed by a one-way analysis of covariance (ANCOVA) adjusted by age, sex and *APOE-ε4.* **P*<0.05, ***P*<0.01, ****P*<0.001, *****P*<0.0001.

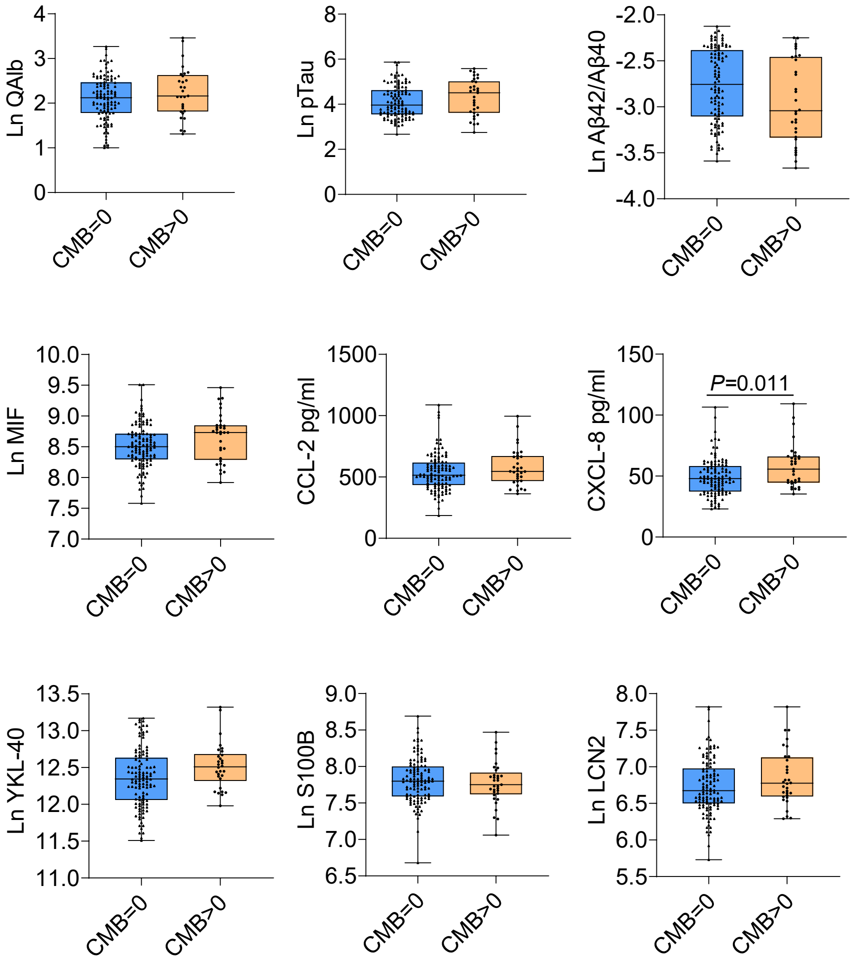

Fig.S4 Levels of fluid biomarkers in different groups defined by CMB (cerebral microbleeds) status. The box plots depict the median (horizontal bar), interquartile range (IQR, hinges), and the whiskers indicate the minimum and maximum values. *P*-values were assessed by a one-way analysis of covariance (ANCOVA) adjusted by age, sex, *APOE-ε4*, Aβ42/Aβ40, and pTau.

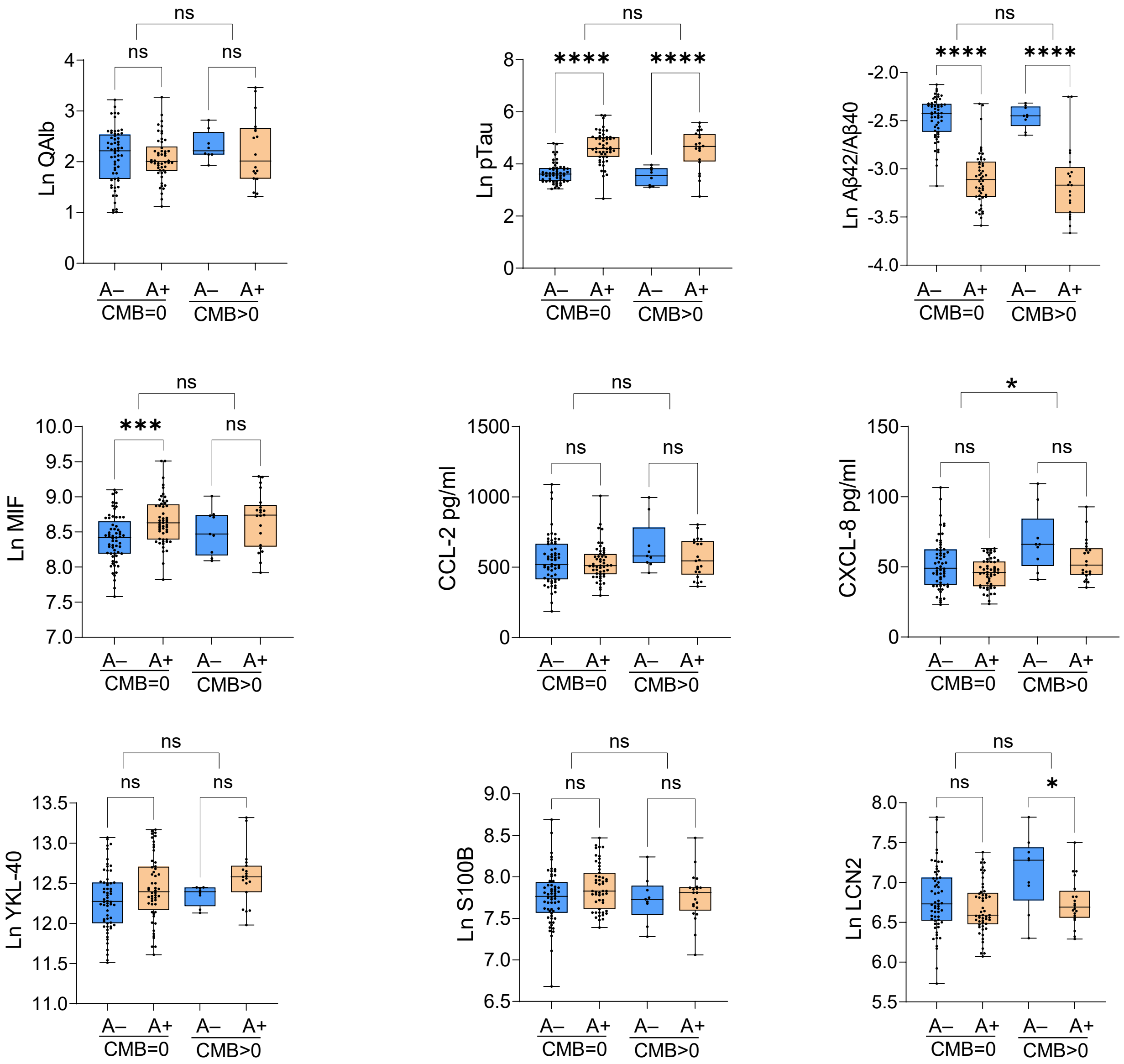

Fig.S5 Levels of fluid biomarkers in different groups defined by Aβ and CMB (cerebral microbleeds) status. The box plots depict the median (horizontal bar), interquartile range (IQR, hinges), and the whiskers indicate the minimum and maximum values. *P*-values were assessed by a one-way analysis of covariance (ANCOVA) adjusted by age, sex and *APOE-ε4.* **P*<0.05, ***P*<0.01, ****P*<0.001, *****P*<0.0001.

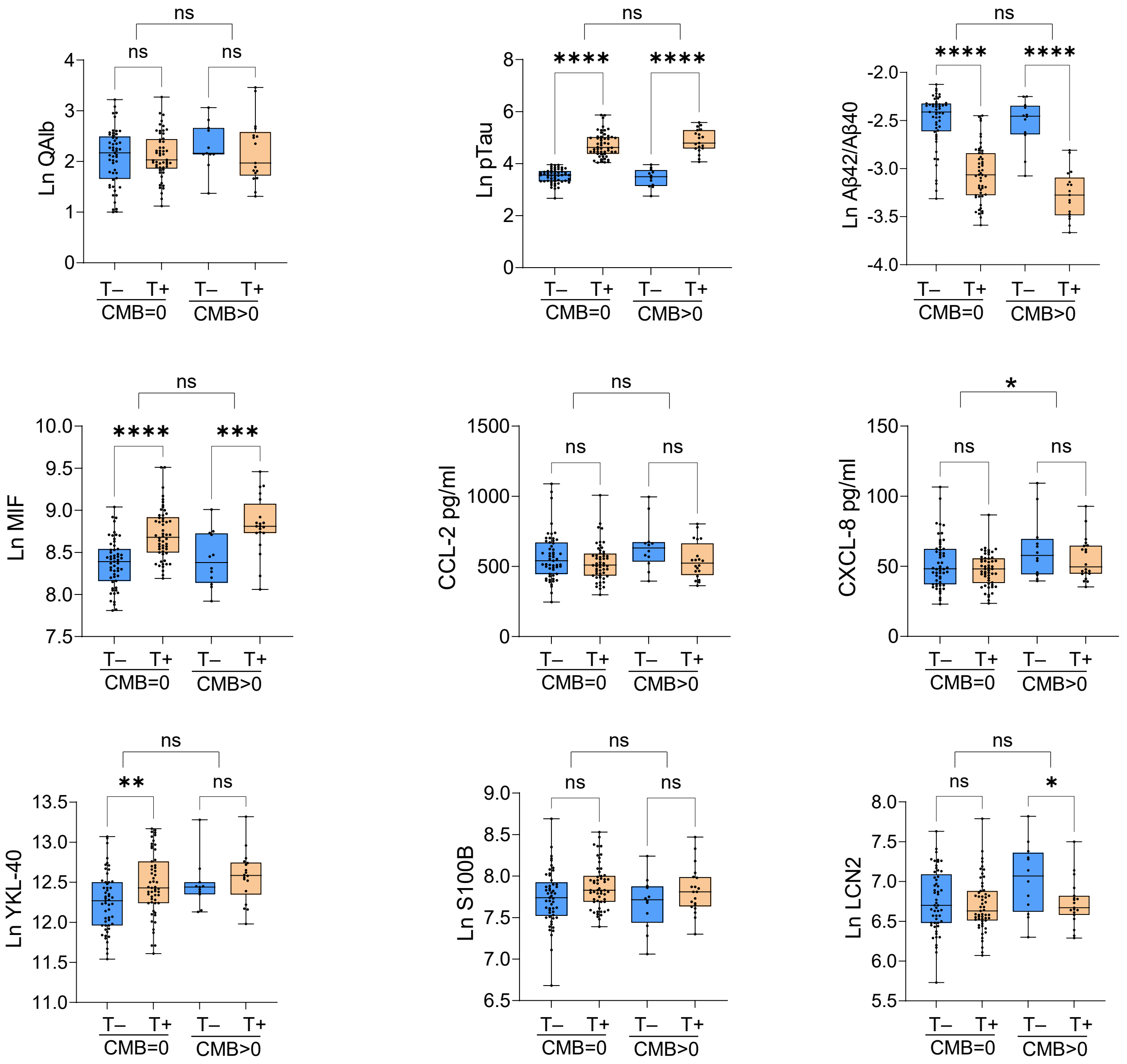

Fig.S6 Levels of fluid biomarkers in different groups defined by tau and CMB (cerebral microbleeds) status. The box plots depict the median (horizontal bar), interquartile range (IQR, hinges), and the whiskers indicate the minimum and maximum values. *P*-values were assessed by a one-way analysis of covariance (ANCOVA) adjusted by age, sex and *APOE-ε4.* **P*<0.05, ***P*<0.01, ****P*<0.001, *****P*<0.0001.

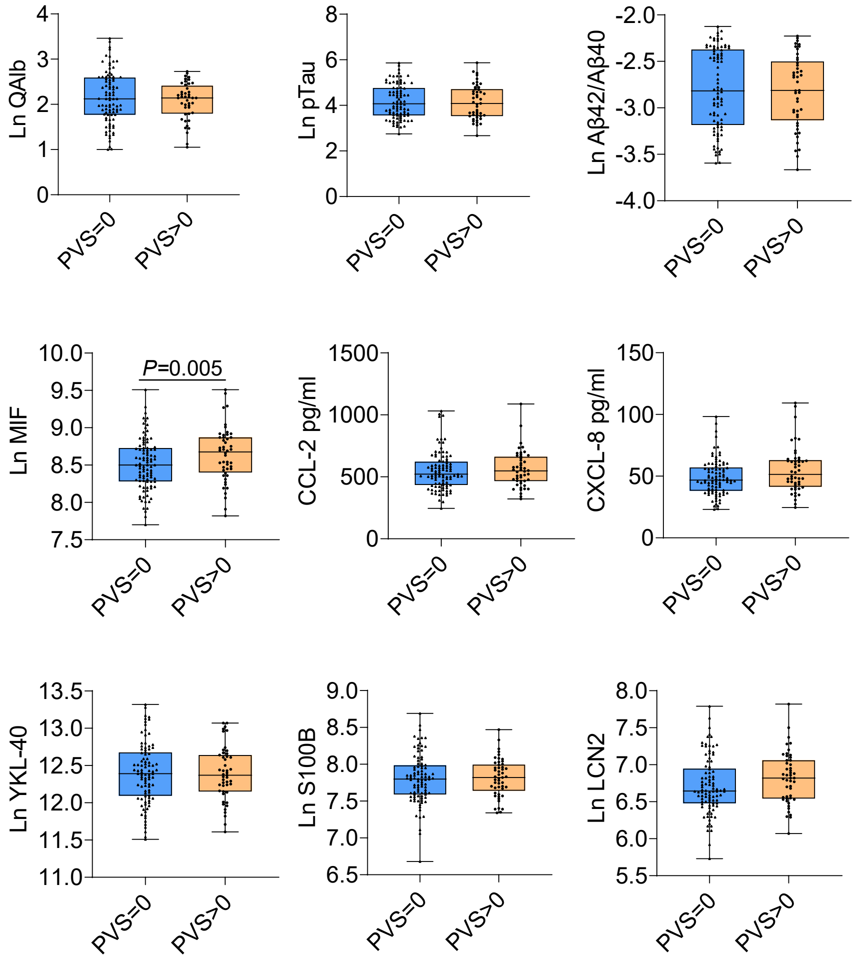

Fig.S7 Levels of fluid biomarkers in different groups defined by PVS (perivascular spaces) status. The box plots depict the median (horizontal bar), interquartile range (IQR, hinges), and the whiskers indicate the minimum and maximum values. *P*-values were assessed by a one-way analysis of covariance (ANCOVA) adjusted by age, sex, *APOE-ε4*, Aβ42/Aβ40, and pTau.

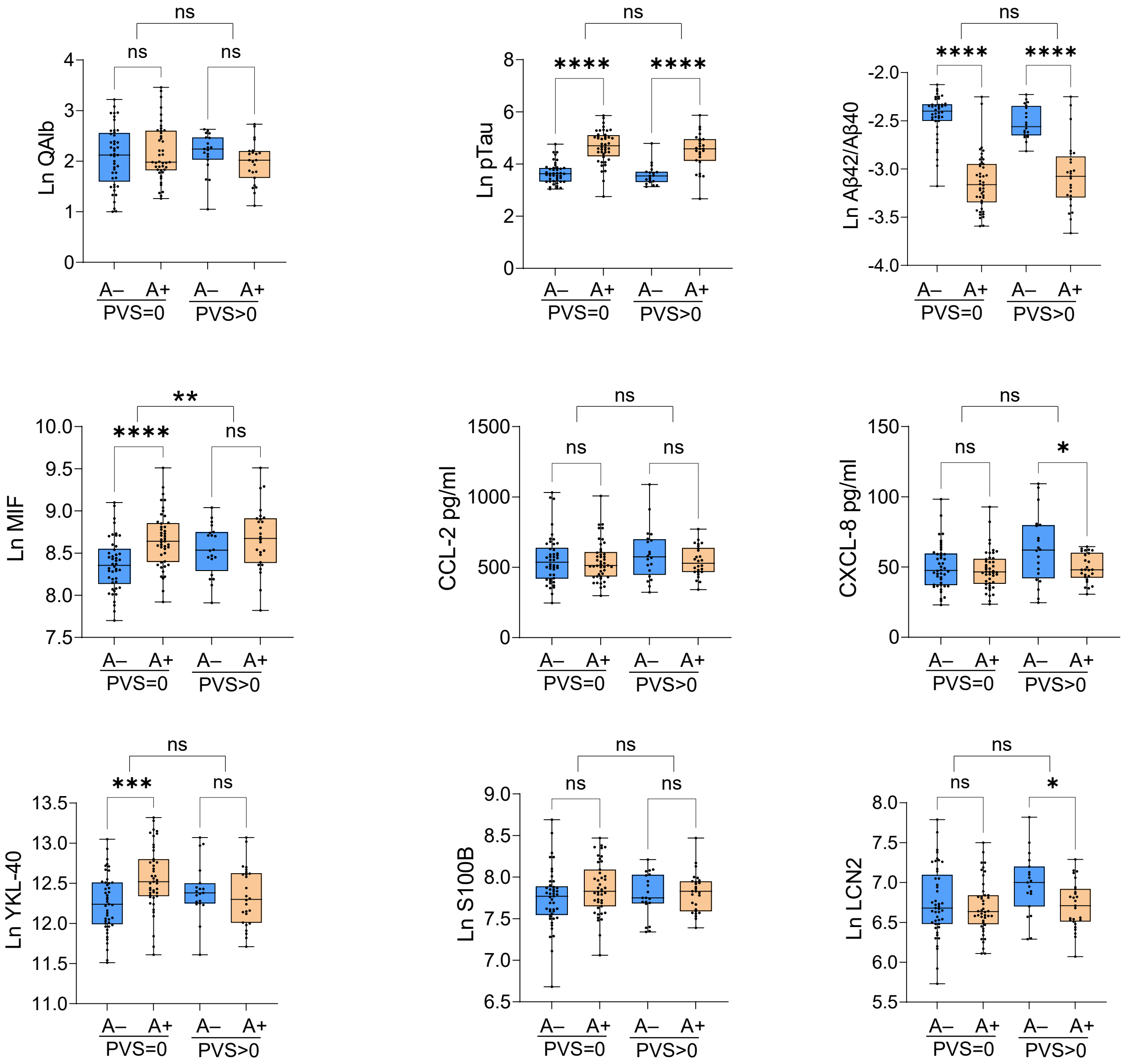

Fig.S8 Levels of fluid biomarkers in different groups defined by Aβ and PVS (perivascular spaces) status. The box plots depict the median (horizontal bar), interquartile range (IQR, hinges), and the whiskers indicate the minimum and maximum values. *P*-values were assessed by a one-way analysis of covariance (ANCOVA) adjusted by age, sex and *APOE-ε4.* *P<0.05, **P<0.01, ***P<0.001, ****P<0.0001.

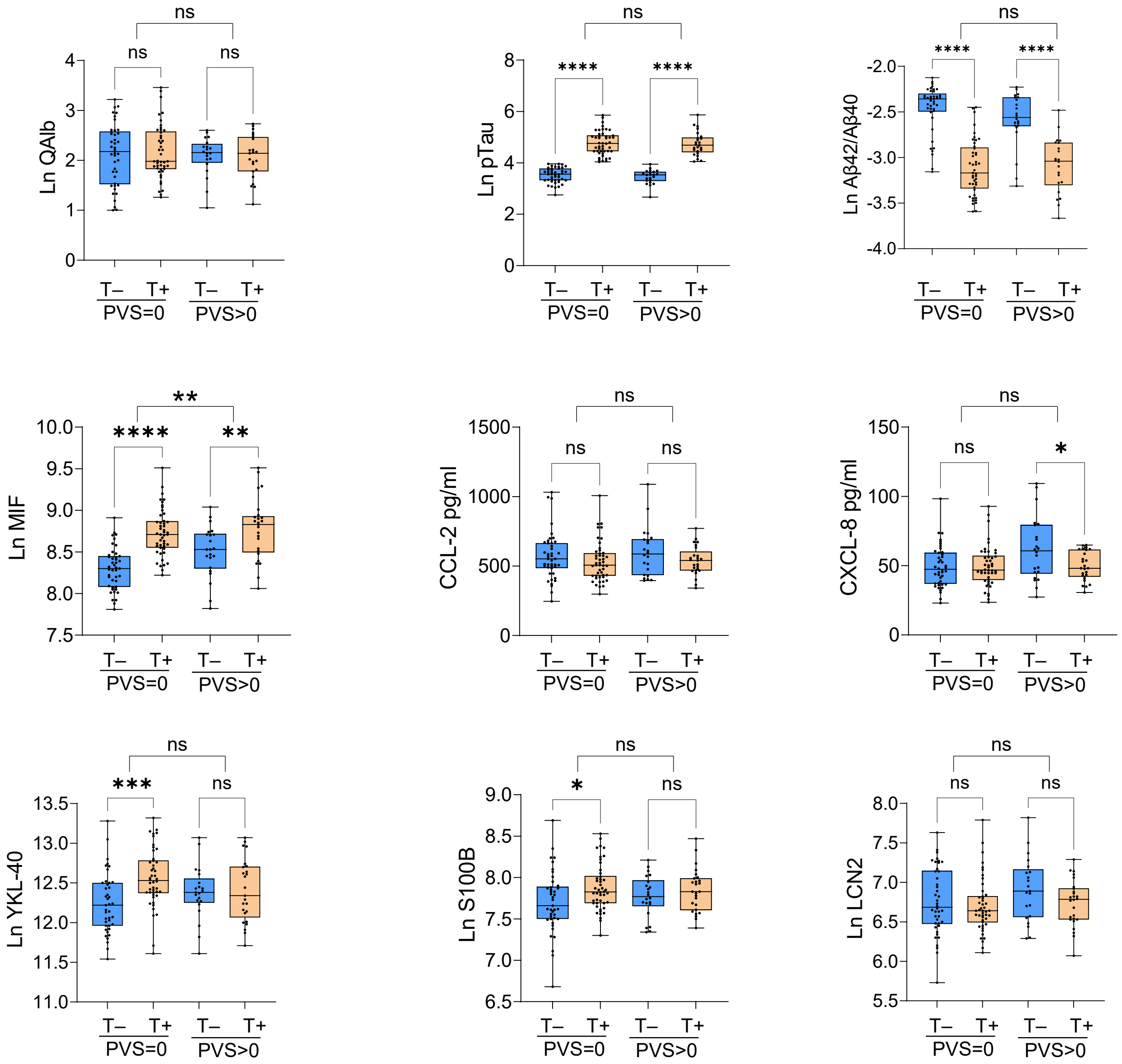

Fig.S9 Levels of fluid biomarkers in different groups defined by tau and PVS (perivascular spaces) status. The box plots depict the median (horizontal bar), interquartile range (IQR, hinges), and the whiskers indicate the minimum and maximum values. *P*-values were assessed by a one-way analysis of covariance (ANCOVA) adjusted by age, sex and *APOE-ε4.* **P*<0.05, ***P*<0.01, ****P*<0.001, *****P*<0.0001.

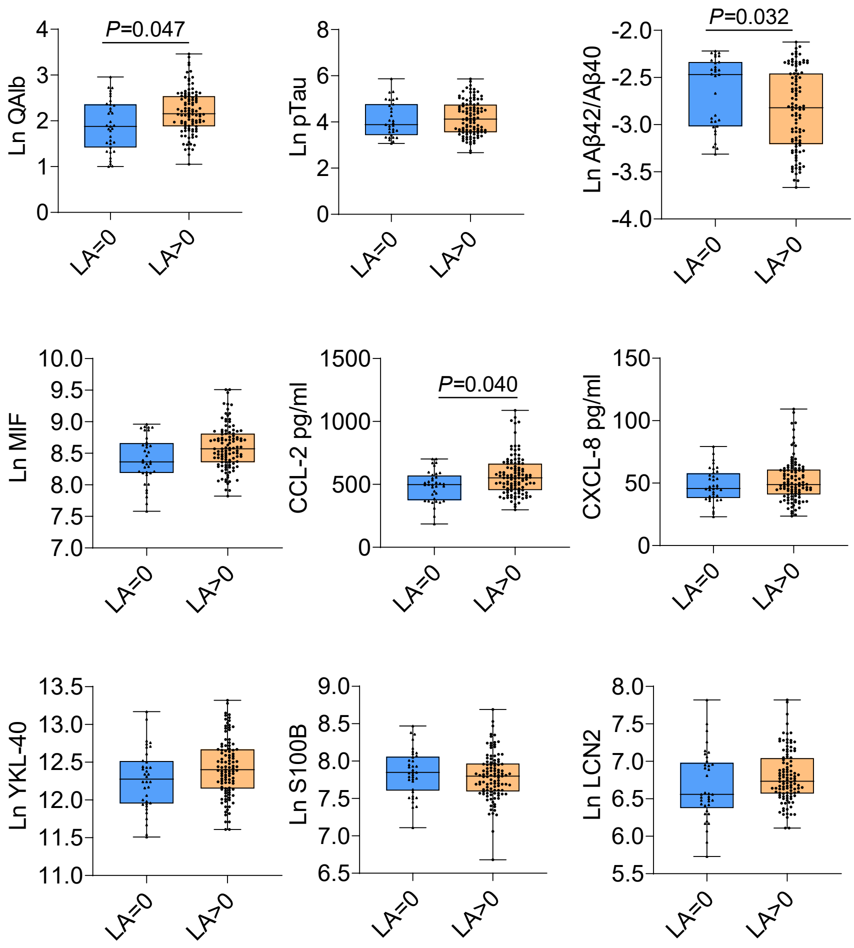

Fig.S10 Levels of fluid biomarkers in different groups defined by LA (lacunes status). The box plots depict the median (horizontal bar), interquartile range (IQR, hinges), and the whiskers indicate the minimum and maximum values. *P*-values were assessed by a one-way analysis of covariance (ANCOVA) adjusted by age, sex, *APOE-ε4*, Aβ42/Aβ40, and pTau.

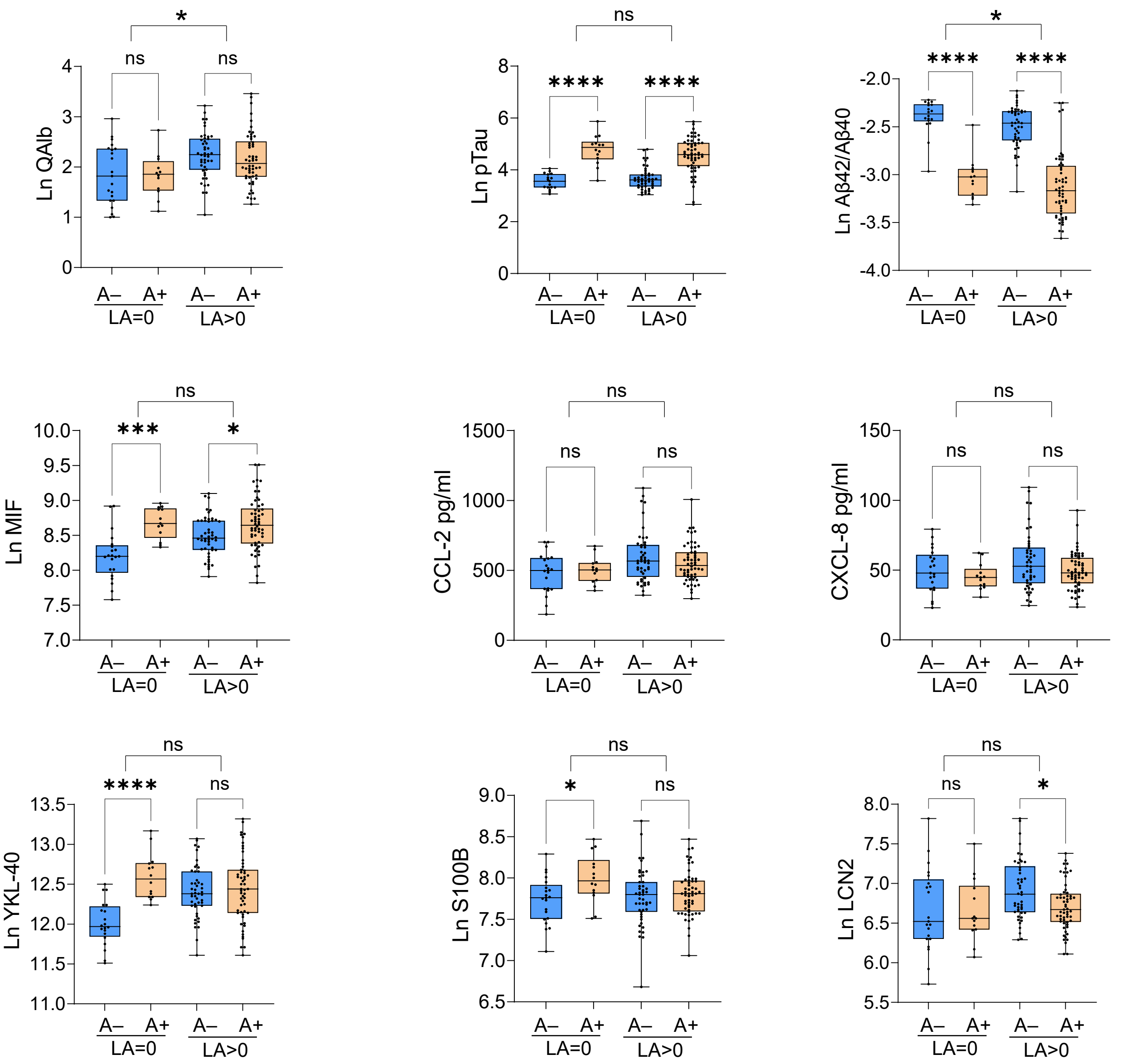

Fig.S11 Levels of fluid biomarkers in different groups defined by Aβ and LA (lacunes status). The box plots depict the median (horizontal bar), interquartile range (IQR, hinges), and the whiskers indicate the minimum and maximum values. *P*-values were assessed by a one-way analysis of covariance (ANCOVA) adjusted by age, sex and *APOE-ε4*. **P*<0.05, ***P*<0.01, ****P*<0.001, *****P*<0.0001.

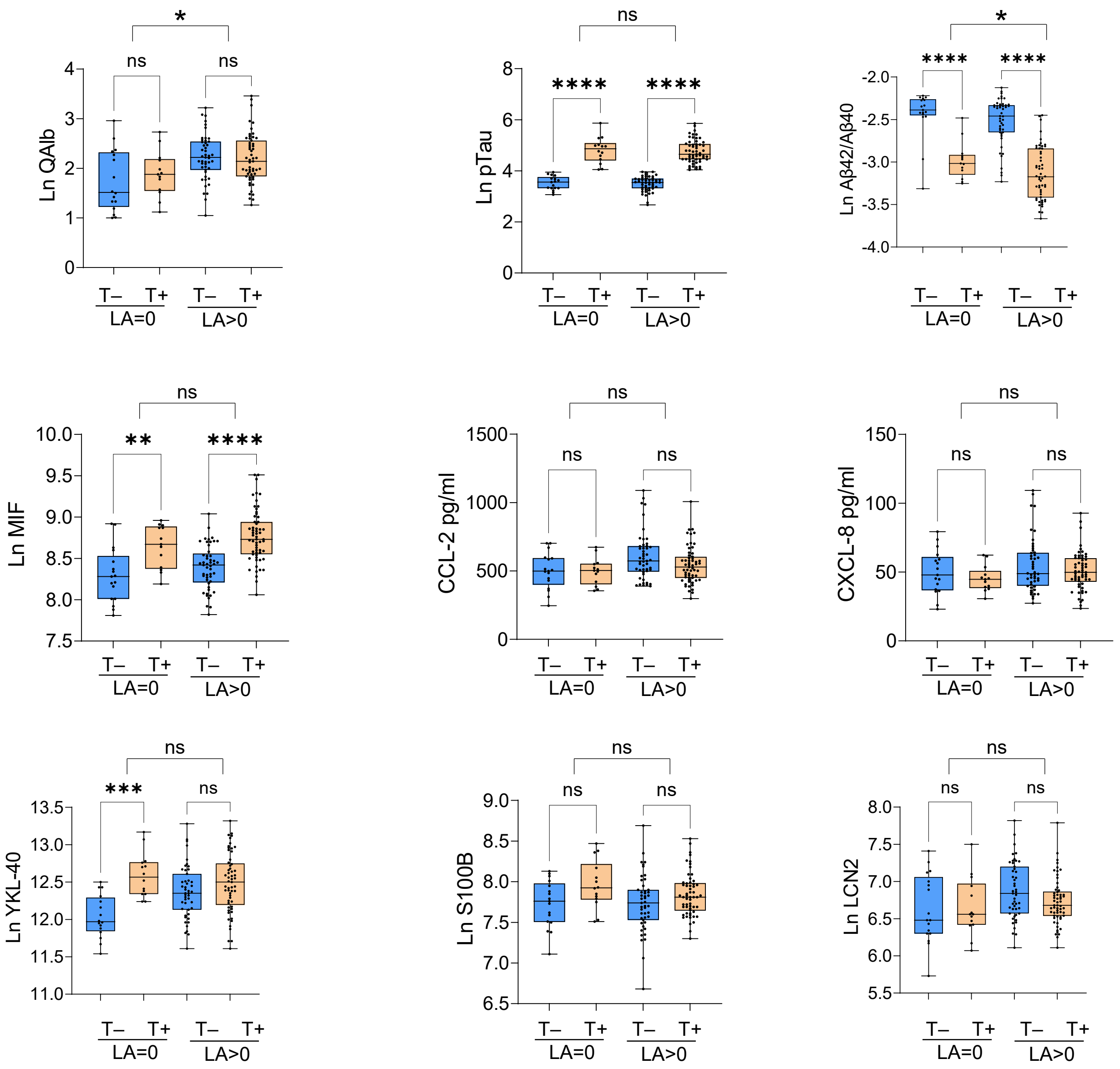

Fig.S12 Levels of fluid biomarkers in different groups defined by tau and LA (lacunes status). The box plots depict the median (horizontal bar), interquartile range (IQR, hinges), and the whiskers indicate the minimum and maximum values. *P*-values were assessed by a one-way analysis of covariance (ANCOVA) adjusted by age, sex and *APOE-ε4.* *P<0.05, **P<0.01, ***P<0.001, ****P<0.0001.

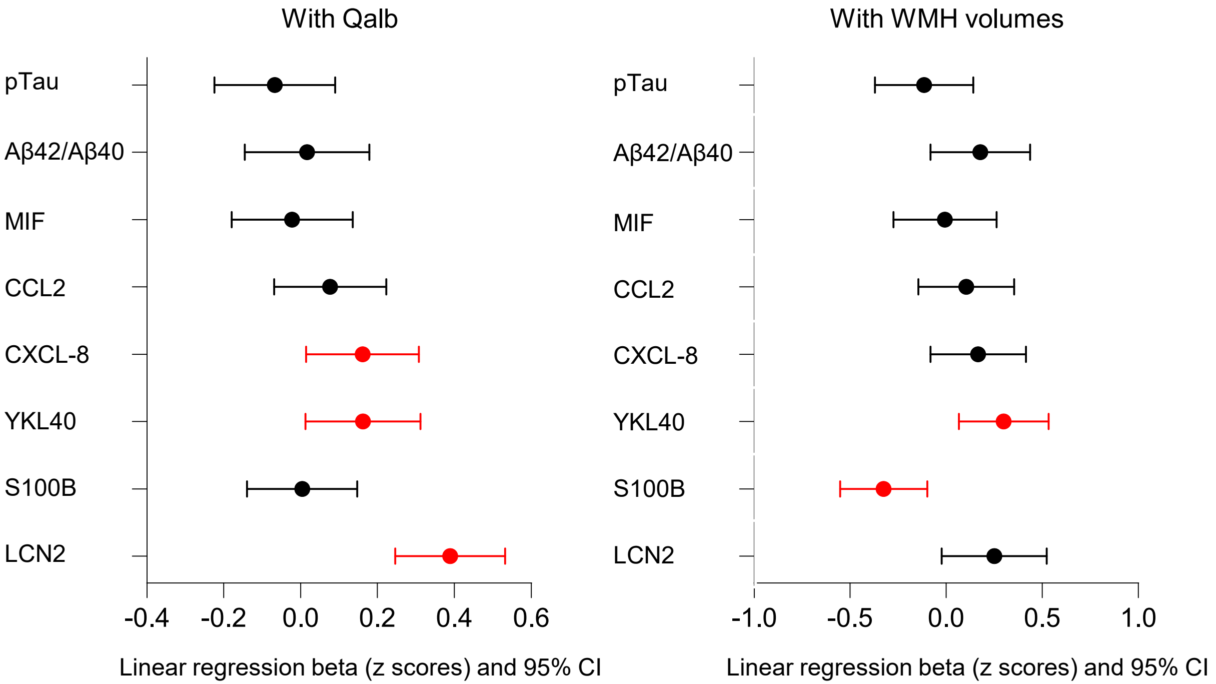

Fig. S13 Associations between CSF neuroinflammatory markers and cerebrovascular damage summarized in forest plots. Linear regression models were adjusted by age, sex, and *APOE-ε4.*

Table S1. Demographic characteristics of the CANDI cohort

		Total (N = 196)	CU (n = 52, 26.%)	MCI (n = 42, 21.4%)	AD (n = 75, 38.3%)	Non-ADD (n = 27, 13.8%)
**Sex, F (%)**		113 (57.7)	25 (48.1)	25 (59.5)	50 (66.7)	13 (48.1)
**Age, years**						
	Mean ± SD	62.8 ± 7.8	60.3 ± 8.7[b,c]	63.9 ± 7.1[a]	64.1 ± 7.3[a]	61.9 ± 7.1
	Median (95% CI)	64.0 (61.7-63.8)	61.0 (57.9-62.8)	66.0 (61.7-66.1)	65.0 (62.4-65.8)	62.0 (59.1-64.7)
**Education, years**						
	Mean ± SD	7.5 ± 4.2	7 ± 3.9	8.9 ± 4.5	7.2 ± 4	7.4 ± 4.2
	Median (95% CI)	8.0 (7.0-8.1)	6.0 (6.0-8.1)	9.0 (7.5-10.3)	6.0 (6.3-8.1)	8.0 (5.7-9.0)
**MMSE**						
	Mean ± SD	18.9 ± 8.1	27.5 ± 2.5[b,c,d]	22.4 ± 4.4[a,c,d]	12.6 ± 6.2[a,b]	14.7 ± 6.7[a,b]
	Median (95% CI)	20.0 (17.8-20.1)	28.0 (26.8-28.2)	22.5 (21-23.7)	13.0 (11.2-14.1)	15.0 (12.1-17.4)
**CDR**						
	Mean ± SD	0.8 ± 0.7	0.0 ± 0.0[b,c,d]	0.5 ± 0.0[a,c,d]	1.5 ± 0.6[a,b]	1.1 ± 0.5[a,b]
	Median (95% CI)	0.5 (0.7-0.9)	0.0 (0.0-0.0)	0.5 (0.5-0.5)	1.0 (1.3-1.6)	1.0 (0.9-1.3)
***APOE-ε*4 carriers, n (%)**		79 (40.3%)	7 (13.5%)[b,c]	19 (45.2%)[a,d]	46 (61.3%)[a,d]	7 (25.9%)[b,c]
**Markers of BBB damage**	QAlb, ratio	9.4 ± 4.9	9.1 ± 4.6	8.9 ± 4.2	9.6 ± 5.7	10.1 ± 4.3
**AD core biomarker**	CSF pTau, pg/mL	80.4 ± 63.7	39.8 ± 16.2[c]	70.2 ± 43[c]	124.6 ± 72.1[a,b,d]	36.1 ± 12[c]
	CSF Aβ42/Aβ40	0.0662 ± 0.0263	0.0902 ± 0.0192[b,c,d]	0.067 ± 0.0266[a,c]	0.0467 ± 0.0155[a,b,d]	0.0744 ± 0.0197[a,c]
**Proinflammatory markers**	CSF MIF, pg/mL	5426.3 ± 2068.1	4210.8 ± 1524.4[c]	5210.4 ± 1616.5[c]	6459.4 ± 2324.6[a,b]	5272.0 ± 1403.5
**Chemokines**	CSF CCL-2, pg/mL	543.1 ± 145.9	518.6 ± 140.2	552.9 ± 163	545.8 ± 125.4	567.6 ± 179.6
	CSF CXCL-8, pg/mL	51.6 ± 15.6	50.3 ± 15.3	46.2 ± 13.6[d]	52.6 ± 13.9	60.1 ± 20.2[b]
**Marker of glial cells**	CSF YKL-40, pg/mL	256458 ± 103572	201916 ± 86081[b,c]	273586 ± 105544[a]	286854 ± 109809[a]	249931 ± 65777
	CSF S100B, pg/mL	2570.1 ± 787.8	2679.2 ± 888.3[d]	2689.1 ± 880.3	2572.1 ± 688.7	2119.2 ± 502.2[a]
	CSF LCN2, pg/mL	934.2 ± 384	1002.6 ± 431.6	838.4 ± 307.7[d]	877.3 ± 314	1112.0 ± 495.5[b]
**CSVD features (%)**	WMH	21 (10.7)	0 (0)	4 (9.5)	11 (14.7)	6 (22.2)
	CMB	33 (16.8)	2 (3.8)	3 (7.1)	21 (28)	7 (25.9)
	PVS	49 (25.0)	2 (3.8)	13 (31)	22 (29.3)	12 (44.4)
	LA	111 (56.6)	20 (38.5)	23 (54.8)	49 (65.3)	19 (70.4)
**CSVD scores**	Mean ± SD	1.4 ± 1.0	0.8 ± 0.8	1.1 ± 0.8	1.8 ± 0.9	2.2 ± 1.1

Categorical variables were evaluated using Pearson's chi-square tests. Comparisons of continuous variables among different groups were assessed by ANCOVA with adjustment for age, sex and *APOE* genotype followed by Bonferroni corrected post-hoc comparisons. $P<0.05$ was considered to indicate statistical significance. [a]Significant values vs CU; [b]Significant values vs MCI; [c]Significant values vs AD; [d]Significant values vs Non-ADD.

Table S2. Associations between features of cerebrovascular damage and neuroinflammation markers

Features		CCL-2	Ln MIF	CXCL-8	Ln YKL-40	Ln S100B	Ln LCN2
**QAlb**	Unstandardized B	0.000	0.037	0.005	0.332	0.021	0.557
	(SE)	0.000	0.155	0.003	0.115	0.136	0.111
	Standardized β	0.125	0.024	0.166	0.238	0.013	0.400
	P value	0.106	0.813	**0.038**	**0.005**	0.875	**<0.0001**
**WMH volumes**	Unstandardized B	17.3	-1161.3	138.2	13806.7	-14459.9	6570.5
	(SE)	14.4	8226.5	116.7	5175.5	5437.6	6171.9
	Standardized β	0.154	-0.024	0.160	0.333	-0.329	0.165
	P value	0.237	0.888	0.241	**0.010**	**0.010**	0.292

Each feature of cerebrovascular damage as a dependent variable was assessed by a linear regression model with CSF neuroinflammation markers as independent variables. The unstandardized regression coefficients (B), standard errors (SE), standardized regression coefficients (β) and *P* values are shown. The model was adjusted for age, sex, *APOE-ε*4, Aβ42/Aβ40, and pTau.

Table S3. Associations between features of cerebrovascular damage and neuroinflammation markers in AD and non-AD (including CU, MCI and Non-ADD) groups.

Group	Features		CCL-2	Ln MIF	CXCL-8	Ln YKL-40	Ln S100B	Ln LCN2
AD	QAlb	Unstandardized B	0.000	-0.425	0.003	0.181	-0.315	0.504
		(SE)	0.001	0.185	0.005	0.168	0.232	0.189
		Standardized β	0.098	-0.279	0.067	0.126	-0.160	0.311
		P value	0.441	**0.025**	0.583	0.286	0.179	**0.009**
	WMH volumes	Unstandardized B	18.7	-8505.3	-23.6	19564.3	-25905.2	14407.1
		(SE)	28.8	10258.9	315.6	7872.6	11524.3	13375.8
		Standardized β	0.112	-0.149	-0.013	0.372	-0.339	0.215
		P value	0.522	0.415	0.941	**0.020**	**0.033**	0.291
Non-AD	QAlb	Unstandardized B	0.000	0.209	0.006	0.354	0.130	0.542
		(SE)	0.000	0.139	0.003	0.132	0.143	0.115
		Standardized β	0.088	0.148	0.216	0.271	0.084	0.439
		P value	0.349	0.137	**0.024**	**0.009**	0.366	**<0.0001**
	WMH volumes	Unstandardized B	5.1	-337.7	214.7	-5615.8	-8370.8	8060.7
		(SE)	10.7	5321.1	63.6	4337.7	3546.1	3625.4
		Standardized β	0.089	-0.012	0.531	-0.245	-0.383	0.411
		P value	0.636	0.950	**0.002**	0.205	**0.025**	**0.034**

Each feature of cerebrovascular damage as a dependent variable was assessed by a linear regression model with CSF neuroinflammation markers as independent variables. The unstandardized regression coefficients (B), standard errors (SE), standardized regression coefficients (β) and *P*-values are shown. The model was adjusted for age, sex and *APOE*-*ε*4.

Table S4. R^2 in models for separate markers significantly associated with QAlb and WMH volumes.

Model (QAlb)	R	R^2	Adjusted R^2	*P* value
**CSF markers + covariates**	0.655	0.429	0.366	**<0.0001**
**Covariates**	0.329	0.108	0.080	**0.003**
**CSF markers**	0.584	0.340	0.314	**<0.0001**
**CXCL8**	0.214	0.046	0.041	**0.004**
**YKL-40**	0.226	0.051	0.046	**0.002**
**LCN2**	0.434	0.188	0.184	**<0.0001**
**Model (WMH volumes)**	**R**	**R^2**	**Adjusted R^2**	***P* value**
**CSF markers + covariates**	0.761	0.578	0.401	**0.016**
**Covariates**	0.406	0.164	0.089	0.071
**CSF markers**	0.631	0.398	0.334	**0.002**
**YKL-40**	0.274	0.075	0.061	**0.025**
**S100B**	0.339	0.115	0.101	**0.005**

Covariates included age, sex, *APOE-ε*4, Aβ42/Aβ40, and pTau.
CSF markers included (1) CXCL-8, YKL-40, and LCN2 in the QAlb model and (2) YKL-40 and S100B in WMH volumes model.
