# Supplementary material for: CSF biomarkers of reactive glial cells are associated with blood–brain barrier leakage and white matter lesions
Source: Transl Neurodegener. 2024 May 23;13:26. doi: 10.1186/s40035-024-00422-z (PMC11112808; doi:10.1186/s40035-024-00422-z)
Supplement: Supplementary file 2 — Additional file 2. Supplementary Methods. [file 40035_2024_422_MOESM2_ESM.pdf]

CSF biomarkers of reactive glial cells are associated with blood–brain barrier leakage and white matter lesions

Supplementary Methods

Study participants

A total of 196 patients as part of the CANDI cohort from the First Affiliated Hospital of University of Science and Technology of China (USTC) were included in the study(1). The CANDI cohort is well characterized in terms of clinical data and fluid biomarkers and contains participants over 45 years of age, including cognitively unimpaired (CU) individuals, patients with mild cognitive impairment, AD dementia, and non-AD dementia (Non-ADD). The inclusion criteria were as follows: participants aged over 45 years who could be accompanied by a reliable partner during clinical visits; were in good general health (with or without cognitive concerns); and were able to undergo clinical tests (including neuroimaging). The exclusion criteria were as follows: patients who had any significant neurological disease other than dementia (including Huntington's disease, seizure disorder, brain tumor and multiple sclerosis); any significant systemic illness or unstable medical condition; or a history of major depression, bipolar disorder, and schizophrenia (that would impact the reliability of cognitive assessments). Individuals who had a Mini-Mental State Examination (MMSE) score >24 and a Clinical Dementia Rating (CDR) score of 0 were defined as CU.

Participants with MCI (CDR = 0.5) preserved activities of daily living, and their complex instrumental functions were either intact or minimally impaired(2). AD patients (CDR≥1) were diagnosed based on the NIA-AA criteria (2011)(3). Patients with non-ADD were those who were diagnosed with dementia but had not been diagnosed with AD, including patients with frontotemporal dementia (FTD, n=8), Lewy body disease (DLB, n=4), vascular dementia (VD, n=7), Parkinson's disease dementia (PDD, n=5), and normal pressure hydrocephalus (NPH, n=3). Participants carrying *ε*4 (heterozygous or homozygous alleles) were defined as *APOE*-*ε*4 carriers.

All patients (or immediate family members in case the patient suffered from dementia) provided written informed consent in this study. This study was conducted according to the Declaration of Helsinki(4) and approved by the ethics committee of the First Affiliated Hospital of the University of Science and Technology China (2019KY-26, 2023KY-117).

CSF biomarker assessment

CSF samples were collected and handled according to a previous study (5). CSF Aβ40, Aβ42, and phosphorylated tau 181 (pTau) levels were measured using commercially available Simoa kits (Quanterix, 101995 and 103714). CSF Aβ and tau status were defined by the CSF Aβ42/Aβ40 ratio and pTau, respectively(6), and the cutoff values were 0.0642 (A+: Aβ42/40<0.0642) for Aβ42/Aβ40 and 54.68 pg/mL for pTau, as described in our previous studies(1). The Luminex platform (Luminex, Luminex 200) and an LXSAHA kit (R&D Systems, LXSAHA-6) were used to measure CSF

concentrations of proinflammatory markers (macrophage migration inhibitory factor [MIF]), chemokines (CCL-2 and CXCL-8), and markers of glial cells (YKL-40, S100B and lipocalin 2 [LCN2]). All samples were assayed in duplicate. For all Simoa analytes, the mean intra-assay coefficient of variation (CV) and inter-CV were ≤2.8% and ≤5.3%, respectively. For Luminex analytes, the mean intra- and inter assay CVs were≤5.0% and ≤9.5%, respectively. All samples were measured above the respective assay lower limits of detection. All these analyses were performed according to the manufacturer's instructions at the Neurodegenerative Disorder Research Centre, USTC, Hefei, China.

Analysis of the CSF/serum albumin quotient (QAlb)

CSF albumin was measured using immunochemical assays (EHALB, Thermo Fisher Scientific), and serum albumin was quantified using a bromocresol green dye binding assay (ADVIA 1800; Siemens, Berlin, Germany). The CSF and serum albumin from paired samples were used to calculate the QAlb using the formula QAlb = [albumin]CSF/[albumin]serum × 1000. Next, QAlb values were stratified by quartiles (Q1: Ln QAlb <1.81; Q2: 1.81< Ln QAlb <2.13; Q3: 2.13< Ln QAlb <2.46; Q4: Ln QAlb >2.46).

Magnetic resonance imaging

Magnetic resonance imaging (MRI) scans of the CANDI cohort were acquired using a GE DISCOVER 750w 3.0T MRI scanner (GE Healthcare, US), including DWI/FLAIR/SWI and T2-weighted images. The total MRI burden of CSVD on an

ordinal scale from 0–4 was determined by counting the presence of the following four features of CSVD[33]: (1) white matter hyperintensities (WMHs, 1 point if present); (2) cerebral microbleeds (CMBs, 1 point if present); (3) perivascular spaces in the basal ganglia (PVSs, 1 point if present); (4) lacunes (LAs, 1 point if ≥ 1 lacune present). Additionality, To further refine our grouping, we merged patients with CSVD scores of 2, 3, and 4 into a single category, denoted as CSVD > 1, representing severe CSVD burden. In contrast, patients with a CSVD score of 1 were categorized as having mild CSVD burden. WMH was defined as increased brightness on T2 images in the brain white matter. The periventricular and deep WMH were evaluated according to the Fazekas rating scale. CMBs were rounded, hypodense lesions with sizes of 2–10 mm in a gradient-recalled echo image or susceptibility-weighted image. PVS was defined as small (< 3 mm) punctate or linear hyperintensities on T2 images, and PVS in the basal ganglia was graded with the semiquantitative rating scale developed by the Edinburg group. LA was defined as a rounded or ovoid lesion of CSF signal measuring 3–20 mm in diameter. Imaging assessment of each CSVD marker was rated by two well-trained raters who were blinded to the participant's clinical data, according to the standards for reporting vascular changes on neuroimaging (STRIVE)(7). Images with inconsistent results were finally assessed by another senior neurologist who was blinded to the initial results. Automated segmentation of WMH was performed using the FMRIB software library v6.0 (FSL; https://fsl.fmrib.ox.ac.uk/fsl/fslwiki) Brain Intensity AbNormality Classification Algorithm (BIANCA) according to the standard protocol [34].

Statistical analyses

Data analyses were performed using SPSS 26.0 (SPSS; Chicago, IL, USA) and GraphPad Prism 9.0 (GraphPad Software; La Jolla, CA, USA). Levels of CSF biomarkers were Ln-transformed if they did not satisfy the normal distribution using the Kolmogorov-Smirnov test. Categorical variables were evaluated using Pearson's chi-square tests. Comparison of CSF biomarkers among different groups defined by CSVD features was assessed by ANCOVA with adjustment for age, sex, *APOE* genotype, Aβ42/Aβ40, and pTau as covariates, followed by Bonferroni-corrected post-hoc comparisons.

The associations between CSF biomarkers and cerebrovascular damage were first analyzed using univariate linear regression models adjusted for age, sex, *APOE*-ε4, Aβ42/Aβ40, and pTau. Then, a multivariable regression analysis was performed, including all univariately significant CSF biomarkers and covariates in the same model. Adjusted R^2 values were used to compare the fit of these regression models. $P<0.05$ was considered to indicate statistical significance.

References

1. Gao F, Lv X, Dai L, Wang Q, Wang P, Cheng Z, et al. A combination model of AD biomarkers revealed by machine learning precisely predicts Alzheimer's dementia: China Aging and Neurodegenerative Initiative (CANDI) study. Alzheimer's & dementia : the journal of the Alzheimer's Association. 2022.
2. Winblad B, Palmer K, Kivipelto M, Jelic V, Fratiglioni L, Wahlund LO, et al. Mild cognitive impairment--beyond controversies, towards a consensus: report of the International Working Group on Mild Cognitive Impairment. Journal of internal medicine. 2004;256(3):240-6.
3. McKhann GM, Knopman DS, Chertkow H, Hyman BT, Jack CR, Jr., Kawas CH, et al. The diagnosis of dementia due to Alzheimer's disease: recommendations from the National Institute on Aging-Alzheimer's Association workgroups on diagnostic guidelines for Alzheimer's disease. Alzheimer's & dementia : the journal of the Alzheimer's Association. 2011;7(3):263-9.
4. World Medical A. World Medical Association Declaration of Helsinki: ethical principles for medical research involving human subjects. Jama. 2013;310(20):2191-4.
5. Dai L, Gao F, Wang Q, Lv X, Cheng Z, Wu Y, et al. Molecules of senescent glial cells differentiate Alzheimer's disease from ageing. Journal of neurology, neurosurgery, and psychiatry. 2023.
6. Jack CR, Jr., Bennett DA, Blennow K, Carrillo MC, Feldman HH, Frisoni GB, et al. A/T/N: An unbiased descriptive classification scheme for Alzheimer disease biomarkers. Neurology. 2016;87(5):539-47.
7. Wardlaw JM, Smith EE, Biessels GJ, Cordonnier C, Fazekas F, Frayne R, et al. Neuroimaging standards for research into small vessel disease and its contribution to ageing and neurodegeneration. The Lancet Neurology. 2013;12(8):822-38.
